# ADP-ribosylation: from molecular mechanisms to human disease

**DOI:** 10.1590/1678-4685-GMB-2019-0075

**Published:** 2019-12-13

**Authors:** Nicolas C. Hoch, Luis M. Polo

**Affiliations:** 1 Departamento de Bioquímica, Instituto de Química, Universidade de São Paulo, São Paulo, SP, Brazil; 2 Cancer Research UK DNA Repair Enzymes Group, Genome Damage and Stability Centre, School of Life Sciences, University of Sussex, Falmer, Brighton, UK; 3 Institute of Histology and Embryology of Mendoza – CONICET, Mendoza, Argentina

**Keywords:** ADP ribose, PARP, DNA damage response, DNA repair, parthanatos

## Abstract

Post-translational modification of proteins by ADP-ribosylation, catalysed by poly (ADP-ribose) polymerases (PARPs) using NAD^+^ as a substrate, plays central roles in DNA damage signalling and repair, modulates a range of cellular signalling cascades and initiates programmed cell death by parthanatos. Here, we present mechanistic aspects of ADP-ribose modification, PARP activation and the cellular functions of ADP-ribose signalling, and discuss how this knowledge is uncovering therapeutic avenues for the treatment of increasingly prevalent human diseases such as cancer, ischaemic damage and neurodegeneration.

## PARP members, structure and activity

ADP-ribosyl transferases, also known as poly(ADP-ribose) polymerases (PARPs), are specific enzymes that transfer the ADP-ribose moiety from β-nicotinamide adenine dinucleotide (NAD^+^) to a target macromolecule, mainly proteins. This activity was identified in the 1960s (Chambon *et al.*, 1963), and almost 20 years later, single- and double-strand DNA breaks were determined as enzyme activators in cell extracts (Benjamin and Gill, 1980a; 1980b). Since then, ADP-ribosylation of proteins has been recognized as a central posttranslational modification in a range of cellular processes, such as DNA damage signalling and repair, transcription, Wnt signalling and programmed cell death (Gibson and Kraus, 2012; Virag, 2013; DaRosa *et al.*, 2015; Kraus, 2015).

**Figure 1:**
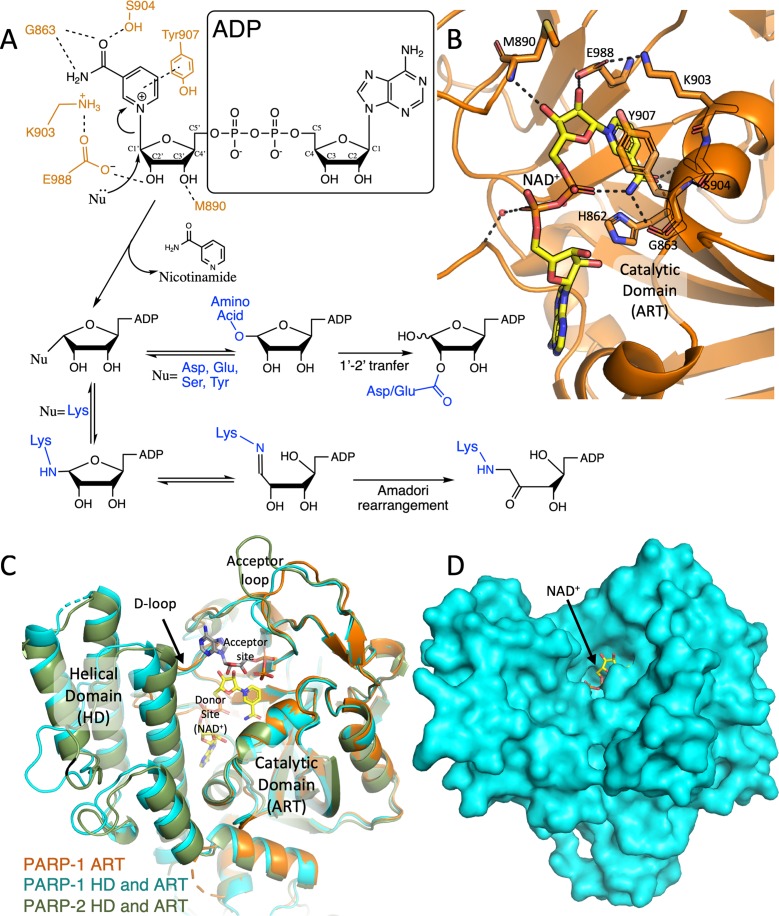
Schematic mechanism of ADP ribosylation reaction and the catalytic domain of DNA-dependant PARPs. A) A simplified overview of the (ADP)-ribosylation reactions catalysed by PARPs. The final products depend on the acceptor residue acting as a nucleophile (Nu, in blue). PARP1 active-site residues interacting with the ribose-nicotinamide moiety of NAD^+^ are illustrated in orange. B) The NAD^+^ (modelled based on the human PARP1 bound to benzamide adenine dinucleotide [PDB: 6BHV], carbon atoms in yellow) in an extended conformation, bound to the catalytic domain of human PARP1 (ART in cartoon, orange, [PDB: 6BHV]). The residues involved in the catalysis are presented as sticks. C) Superposed cartoon view of human PARP-1 ART domain (orange, [PDB: 6BHV]), PARP1 (light blue, [PDB: 5WS1]) and PARP2 (green, [PDB: 3KJD]) showing the structure of the entire catalytic domains (ART and HD). The modelled NAD^+^ (in yellow) denotes the donor site, while a molecule of ADP (modelled by superimposing the structures of chicken PARP1 [PDB: 1A26] to the human PARP1 [PDB: 3KJD]) indicates the acceptor site. Donor loop (D-loop) and acceptor loop are labelled. D) Surface representation of human PARP1 [PDB: 3KJD] with NAD^+^ modelled into the active site. The ribose group to be attacked is exposed to the solvent.

ADP-ribosylation can occur either as a single mono(ADP-ribose) unit (MAR) or as poly(ADP-ribose) (PAR) chains, which can be linear or branched. Since the ADP-ribosyl (ADPr) group contains a high density of negative charges, the addition of ADPr units can dramatically change the biophysical properties of a target protein or promote protein-protein interactions (Figure 1). For example, long PAR chains have been proposed to produce a halo of negatively charged density around the target protein, disrupting the liquid phase in which the protein is embedded (Altmeyer *et al.*, 2015).

There are 17 known members of the PARP family in the human genome (Barkauskaite *et al.*, 2015), and most of these possess the ability to auto-modify, often on multiple sites (Vyas *et al.*, 2014). However, only a few are *bona fide* poly(ADP-ribose) polymerases, while most are in fact mono(ADP-ribosyl) transferases (Vyas *et al.*, 2014). In human cells, the majority of PARP activity is exerted by PARP1 (85%-90%) and by PARP2 (10%–15%) (Szanto *et al.*, 2012).

PARPs are multidomain proteins that contain a common structurally related catalytic domain that is also found in a range of pathogenic toxins from both gram-positive and gram-negative bacteria such as *Bacillus sphaericus*, *Clostridium sp.*, *Corynebacterium diphteriae*, *Salmonella enterica, Vibrio cholera* and *Escherichia coli*, (Laing *et al.*, 2011; Karlberg *et al.*, 2013; Simon *et al.*, 2014; Barkauskaite *et al.*, 2015; Langelier *et al.*, 2018). The catalytic domains of 5 of the 17 human members – PARP1, 2, 3, 4 and 16 –, contain an additional subdomain known as helical domain (HD), which has autoinhibitory functions by sterically hindering NAD+ binding and has to be removed for every catalytic cycle (Dawicki-McKenna *et al.*, 2015; Langelier *et al.*, 2018).

### Reaction mechanism

The PARP1 active site is formed between the catalytic domain (ART domain) and the helical domain (HD) (Figure 1B, C). The substrate to be PARylated binds to the acceptor site on the surface of the ART domain, defined by the acceptor loop (residues 977 to 988 in PARP1) that is also thought to regulate polymer length and chain branching (Vyas *et al.*, 2014; Chen *et al.*, 2018). NAD^+^ binds to the donor site in an extended conformation, such that the ADP-ribose moiety interacts with the D-loop in the ART domain (residues 875 to 894 in PARP1) (Gibson and Kraus, 2017), while the nicotinamide moiety forms three hydrogen bonds with Gly863, Ser904 and Tyr907 (PARP1 numbering) (Figure 1B, C) (Langelier *et al.*, 2018).

Two reaction mechanisms have been proposed, with detailed structural evidence supporting the second mechanism (Tsurumura *et al.*, 2013). One is an S_N_2 displacement mechanism, with the formation of a penta-coordinated transition state (Marsischky *et al.*, 1995), while the other is an S_N_1 strain-alleviation mechanism that involves the formation of a stable furanosyl oxocarbenium ion (van Rijssel *et al.*, 2014), and a rotation around the phosphodiester bond (Simon *et al.*, 2014; Cohen and Chang, 2018). In either case, the nucleophilic attack is performed by an oxygen or a nitrogen atom from the side chain of the target amino acid, which can be glutamic acid, aspartic acid, serine, cysteine, arginine, lysine or tyrosine (Ogata *et al.*, 1980; Altmeyer *et al.*, 2009; Laing *et al.*, 2011; Rosenthal and Hottiger, 2014; Bonfiglio *et al.*, 2017; Leslie Pedrioli *et al.*, 2018). Subsequently, the product can have chemical reorganisations: glutamate and aspartate modifications undergo a C1’–C2’ transfer, and lysine linkages suffer an Amadori rearrangement to form a stable ketoamine (Altmeyer *et al.*, 2009; Morgan and Cohen, 2015; Cohen and Chang, 2018) (Figure 1A). Ultimately, nicotinamide is released as a by-product. Linear PAR chains are formed using the hydroxyl group in C2 of the ADP-ribose moiety, and branching involves the oxygen in C2’ for the nucleophilic attack (Juarez-Salinas *et al.*, 1982; Chen *et al.*, 2018).

Recently, an important modifier of PARP catalytic activity, termed histone PARylation factor (HPF1), was described (Gibbs-Seymour *et al.*, 2016). HPF1 is responsible for switching specificity of PARP1/2 towards serine and tyrosine residues and from auto-PARylation to PARylation of chromatin components and remodellers (Bonfiglio *et al.*, 2017; Leslie Pedrioli *et al.*, 2018). HPF1 also seems to modulate the length of ADPr polymers and can itself be mono(ADP)ribosylated by PARP1 (Leslie Pedrioli *et al.*, 2018). Recent studies revealed that serine could be the predominant PARylation site at chromatin after DNA damage (Leidecker *et al.*, 2016; Palazzo *et al.*, 2018). Strikingly, some results indicate that despite the presence of a hydroxyl group and the resemblance with serine, threonine is not modified by PARP in mammalian cells (Leslie Pedrioli *et al.*, 2018).

### Domain architecture and activation

In addition to the catalytic domain, PARPs contain different domains that mediate protein-protein or protein-nucleic acid interactions, such as ankyrin repeats (PARP5a and 5b, called tankyrases); CCCH zinc fingers (PARP7, 12 and 13), and macrodomains (PARP9, 14 and 15) (Gibson and Kraus, 2012; Karlberg *et al.*, 2013; Barkauskaite *et al.*, 2015). The DNA-dependent PARPs 1, 2 and 3 have DNA binding domains that promote their activation by DNA breaks. These proteins contain a WGR (Trp-Gly-Arg) domain, which upon DNA binding promotes conformational changes in the HD that activate the catalytic domain (Eustermann *et al.*, 2015; Grundy *et al.*, 2016; Obaji *et al.*, 2018).

In PARP1, the WGR domain is not involved in the initial recognition and binding of DNA-breaks (Eustermann *et al.*, 2015). Instead, three zinc fingers (ZnFs) make the primary contact with the DNA. The first two ZnFs at the PARP1 N-terminus are necessary and sufficient for protein recruitment to DNA-damage sites *in vivo*, using structurally equivalent residues (Ali *et al.*, 2012). Recent NMR studies suggest that ZnF2 is the leading domain that binds to the 3’ end of the break, followed by ZnF1, which recognises the 5’ end. This complex promotes ZnF3 recruitment, which leads to WGR domain binding to a surface formed by ZnF1, ZnF3 and DNA (Eustermann *et al.*, 2015). Interestingly, PARP1 makes much more extensive contacts with the DNA surrounding the break than at the break site *per se*, allowing for the recognition of DNA breaks from a variety of sources. In contrast, the WGR domains of both PARP2 and PARP3 (which do not have ZnFs) play a key role in DNA binding and discriminate between different DNA ends by recognising the presence of a 5’phosphate group at the DNA break site (Langelier *et al.*, 2014; Grundy *et al.*, 2016; Obaji *et al.*, 2018).

In addition to the domains involved in DNA-break recognition and catalytic activation, PARP1 contains a BRCT-like (BRCA1 C-terminus) domain where most of the auto-modification sites have been identified (Altmeyer *et al.*, 2009; Tao *et al.*, 2009) and which is implicated in mediating protein-protein interactions (Liu *et al.*, 2011; Noren Hooten *et al.*, 2011; Hsu *et al.*, 2019)

### ADP-ribosylation of DNA

ADP-ribosylation was long considered a protein modification exclusively. However, recent reports have independently shown that DNA-dependent PARPs can add ADPr covalently to DNA ends, at least *in vitro* (Talhaoui *et al.*, 2016; Munnur and Ahel, 2017; Zarkovic *et al.*, 2018).

PARP1, PARP2 and PARP3 all modify both 3’ or 5’ terminal phosphate groups via a phosphodiester bond, and PARP1 and PARP2 can also modify free 3’hydroxyl groups to generate a ribose-ribose bond (Talhaoui *et al.*, 2016; Munnur and Ahel, 2017; Zarkovic *et al.*, 2018). Modification of 5’ phosphorylated ends may protect them from phosphatase activity, offering a possible function for this modification *in vivo*. Surprisingly, ADP-ribosylation of single-stranded DNA gaps promoted their ligation by DNA ligases even in the absence of ATP, suggesting that DNA modification “activates” these ends for ligation (Belousova *et al.*, 2018). However, it is currently unclear if and how this promotes DNA repair *in vivo*.

## Cellular Functions of ADP-ribosylation

### DNA damage signalling and repair

Perhaps the best-studied cellular role of ADP-ribosylation is the crucial function of PARP1 and PARP2 in promoting the repair of DNA strand breaks (Ray Chaudhuri and Nussenzweig, 2017). PARP1 is a sensor of DNA breaks with high affinity for DNA and a lesion recognition mechanism that allows it to be activated by DNA breaks induced by a broad range of sources (Eustermann *et al.*, 2015).

PARP1 activation leads to extensive HPF1-assisted PARylation of chromatin components surrounding DNA damage sites (Boulikas, 1988; Gibbs-Seymour *et al.*, 2016). PARylation of histone H1 and all four nucleosomal histones, as well as HMG proteins, occurs on a number of modification sites, predominantly serines (Bonfiglio *et al.*, 2017; Palazzo *et al.*, 2018), but whether these have differing functions or are simply a chromatin attachment site for PAR chains is currently unclear. In addition to changes in the chromatin environment (discussed below), PARylation leads to the recruitment of a myriad of DNA repair factors, which often contain dedicated PAR-binding domains such as BRCT, PBZ, WWE and macrodomain, or a short positively charged peptide sequence termed the PAR-binding motif (PBM) (Beck *et al.*, 2014a). Crucially, PARP1 auto-modification reduces its affinity for DNA, allowing the repair machinery to access the damage site (Satoh and Lindahl, 1992).

**Figure 2:**
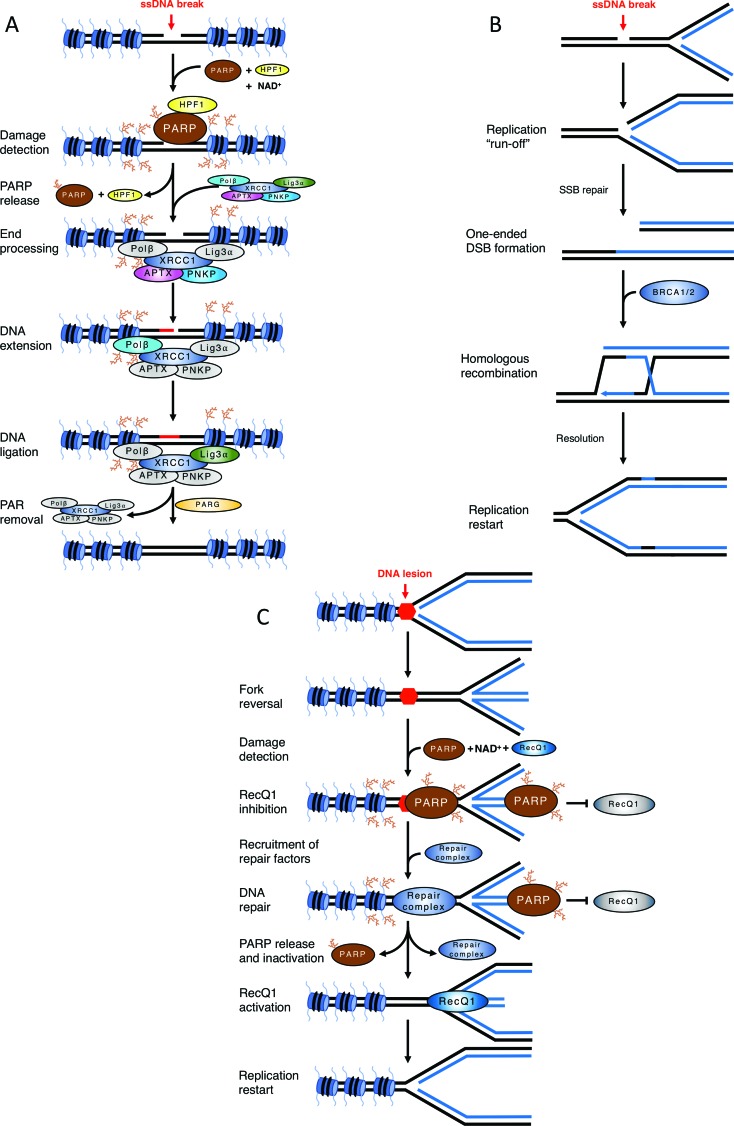
Examples of the impact of ADP-ribosylation in DNA damage signalling and repair. A) Mechanism of single-strand break repair. A single-strand break activates PARP1/2, leading to HPF-1 assisted PARylation of chromatin. PARP auto-modification causes its release and PAR chains surrounding the break site recruit XRCC1 complex. APTX and PNKP process break termini, Polβ fills the gap by DNA synthesis and DNA Ligase IIIα seals the remaining nick. PARG removes PAR chains and XRCC1 complex is released, completing the repair. B) Defective single-strand break repair causes a reliance on homologous recombination. An unrepaired single-strand break is encountered by an ongoing replication fork, which converts it into a one-ended double-strand break. This lesion is repaired by BRCA1 and BRCA2-dependent homologous recombination. C) Role of PARP1/2 in fork reversal. A replication fork encounters an obstacle to its progression and reverts. PARP is activated either by the obstacle/lesion itself or by the DNA end at the regressed fork. PAR chains prevent RecQ1 binding/activity. Upon resolution of the block, PARP release (and presumably PAR chain degradation by PARG) allow RecQ1 helicase to access the reversed fork and remodel it back into a canonical replication fork.

In the case of DNA single-strand break repair (SSBR), PARP1 and PARP2-dependent ADP-ribosylation leads to the recruitment of the central scaffolding protein XRCC1, which contains a PAR-binding BRCT domain (Caldecott, 2008; Breslin *et al.*, 2015; Polo *et al.*, 2019). Lesions repaired by this pathway arise predominantly from oxidative damage to the DNA, but are also formed as intermediates of the base excision repair pathway or by the abortive activity of topoisomerases and DNA ligases (Caldecott, 2014). XRCC1 interacts with DNA and a range of DNA modifying enzymes that process these lesions to restore canonical 3’OH and 5’P termini required for subsequent re-ligation of the damaged strand by DNA ligase III (Caldecott, 2008; Polo *et al.*, 2019) (Figure 2A).

In cycling cells, this pathway prevents the collision of unrepaired single-strand breaks (SSBs) with the DNA replication machinery, which would convert SSBs into much more deleterious DNA double-strand breaks (DSBs) (Figure 2B). As these DNA replication-induced DSBs are one-ended, their accurate repair requires homologous recombination using the sister chromatid (Saleh-Gohari *et al.*, 2005; Cortes-Ledesma and Aguilera, 2006). This leads to a distinctive requirement for functional homologous recombination in cells with defective single-strand break repair, as discussed in the context of PARP inhibitors below. Interestingly, SSBR was recently shown to serve as a backup mechanism for the “repair” of unligated Okazaki fragments during DNA replication (Hanzlikova *et al.*, 2018) and is also thought to play a role in a sub-pathway of DNA double-strand break repair termed microhomology-mediated end-joining (Sfeir and Symington, 2015).

PARP1 also plays a crucial role in promoting the reversal of dysfunctional DNA replication forks (Ray Chaudhuri *et al.*, 2012; Ray Chaudhuri and Nussenzweig, 2017). Fork reversal is an active process that occurs when DNA replication stalls due to impediments to the progression of the replisome (Zellweger *et al.*, 2015) and involves the formation of a “chicken foot” structure in which the newly synthesised daughter strands anneal to each other (Quinet *et al.*, 2017) (Figure 2C). The molecular mechanisms of this process are currently under intense investigation, but PARP1 seems to stabilise reversed forks by preventing the helicase RECQ1 from dismantling the reversed DNA arm (Berti *et al.*, 2013).

PARP1 engagement of DNA breaks, particularly DSBs, has to be carefully coordinated with other end-binding proteins to ensure genomic stability. The Ku70/Ku80 heterodimer is a sensor of DSBs for repair by the non-homologous end-joining pathway (NHEJ) (Shibata *et al.*, 2018). PARP1 is thought to compete with Ku for DSB binding so that PARP1 loss allows Ku to engage DNA ends aberrantly and vice-versa, leading to damage hypersensitivity and genomic instability (Hochegger *et al.*, 2006; Cheng *et al.*, 2011). This is highlighted by a recent report suggesting that PARP1 may participate in the eviction of Ku from breaks that are destined for repair by NHEJ-independent pathways (Yang *et al.*, 2018). Conversely, the Mre11/Rad50/Nbs1 (MRN) complex, which also plays a very early role in the signalling and repair of DSBs, has been suggested to require PARP1 for its recruitment to break sites (Haince *et al.*, 2008; Bryant *et al.*, 2009). However, there are also instances in which PARP1 activation must be actively suppressed, such as at telomeres, where the shelterin complex, and in particular TRF2, prevents PARP1 binding to avoid attempts of “repairing” telomeric DNA ends (Schmutz *et al.*, 2017).

PARP1 and PARP2 are partially redundant, as illustrated by the early embryonic lethality of the double knockout mouse (Menissier de Murcia *et al.*, 2003). Phenotypically, PARP2 can replace PARP1 for many of the roles described above, but is restricted in part by a more limited specificity for DNA breaks with 5’P ends (Langelier *et al.*, 2014). Both enzymes are redundant for XRCC1 recruitment to oxidative lesions (Hanzlikova *et al.*, 2017) and the repair of DNA base damage (Ronson *et al.*, 2018), but only PARP1 seems to generate ADP-ribose in response to topoisomerase poisons (Hoch *et al.*, 2017). Surprisingly, PARP2 seems unable to modify the same target sites as PARP1, suggesting that this redundancy is indirect (Leslie Pedrioli *et al.*, 2018). Intriguingly, a recent study suggested that PARP2 extends PARP1-generated PAR chains, introducing branching points that are recognised by branching-specific factors (Chen *et al.*, 2018).

The other DNA-dependent ADP-ribosyl transferase PARP3, although activated by DNA breaks *in vitro*, has less clear roles in DNA repair, and has been implicated in double-strand break (DSB) repair by non-homologous end-joining (Rulten *et al.*, 2011), particularly during IgG class switching (Robert *et al.*, 2015), regulation of DSB repair pathway choice (Beck *et al.*, 2014b) and most recently the repair of G4-quadruplex containing DNA lesions (Day *et al.*, 2017; Layer *et al.*, 2018).

ADP-ribosylation also controls telomere length. TRF1, a telomere-binding protein, is PARylated by the tankyrase PARP5a (or TNKS1), which reduces its affinity for the telomere and allows telomerase to access the DNA end for elongation (Smith *et al.*, 1998). Similarly, PARP2 has been shown to contribute to telomere homeostasis by modifying TRF2 (Dantzer *et al.*, 2004). Other PARPs, such as PARP9, PARP10 and PARP15 also play roles in DNA repair (Yan *et al.*, 2013; Nicolae *et al.*, 2014; Nicolae *et al.*, 2015), suggesting that the interplay between ADP-ribosylation and genomic stability may be even more extensive than currently known.

### DNA-dependent PARPs and chromatin

PARP1 can be thought of as an integral component of chromatin that modifies chromatin structure directly (Clark *et al.*, 2012). For example, PARP1 was shown to compete with histone H1 for binding to linker DNA (Poirier *et al.*, 1982; Kim *et al.*, 2004) and is reported to have intrinsic histone chaperone activity *in vitro*, mediated in part by the highly negatively charged nature of the PAR polymer (Muthurajan *et al.*, 2014). This is further illustrated by the extensive PARP1-dependent modification of core and linker histones (Boulikas, 1988), as well as the existence of H2A variants with PAR-binding domains that may well mediate long-range PARP-dependent chromatin interactions (Timinszky *et al.*, 2009).

PARP1 also regulates chromatin accessibility indirectly by recruiting chromatin remodellers, such as ALC1, SMARCA5 and CHD2 (Ahel *et al.*, 2009; Smeenk *et al.*, 2013; Luijsterburg *et al.*, 2016). Interestingly, processing of PARP1-generated PAR chains by PARG and NUDIX5 hydrolases has been suggested to provide a localised pool of ATP in the nucleus for ATP-consuming chromatin remodelling complexes (Wright *et al.*, 2016). PARP1 activation at gene promoters also controls the induction of transcription, such as chromatin “puffing” of heat shock-inducible genes in *Drosophila* polytene chromosomes (Tulin and Spradling, 2003), and at gene promoters responsive to transcription factors such as NFκB, PPARγ and hormone receptors such as ER, AR and RAR (Kraus and Hottiger, 2013). Recently, PARP1 has also been linked with the regulation of RNA polymerase II pausing via the negative elongation factor NELF-E (Gibson *et al.*, 2016). However, a more precise understanding of the molecular mechanisms involved in most of these processes and reconciliation with the fact that PARP1 KO mice have very mild phenotypes are still lacking.

Although the partial redundancy between PARP1 and PARP2 is clear for DNA damage-related functions, whether this extends to chromatin remodelling and transcriptional regulation is unclear. A screen for PARP2 targets revealed an enrichment of proteins associated with transcriptional regulation and RNA splicing, suggesting this might be the case. Similarly, targets of PARP3 were enriched in RNA processing, transcription and chromatin organization (Bartolomei *et al.*, 2016), suggesting that all three DNA-dependent PARPs may well be involved in important DNA-damage independent aspects of chromatin biology.

### ADP-ribose in cellular signalling

ADP-ribosylation is also involved in controlling several signalling cascades, such as Wnt/β-catenin, NFκB and the unfolded protein response. The two tankyrases PARP5a and PARP5b (TNKS1 and TNKS2) PARylate axin, a central component in the β-catenin destruction complex, leading to its proteasomal degradation via RNF146, a PAR-dependent E3 ubiquitin ligase (Huang *et al.*, 2009). Wnt signalling is further promoted by PARP10-dependent mono-ADP-ribosylation of GSK3β, which inhibits its kinase activity and also stabilises β-catenin (Feijs *et al.*, 2013). PARP10 additionally suppresses NFκB signalling via MARylation and inactivation of NEMO (Verheugd *et al.*, 2013), and PARP16 was shown to MARylate and activate PERK and IRE1α, central signalling hubs in the unfolded protein response in the endoplasmic reticulum (Jwa and Chang, 2012).

Many PARPs are involved in cellular antiviral mechanisms, with PARP7, PARP9, PARP12 and PARP14 all implicated in the interferon response, and PARP13 is involved in direct degradation of viral transcripts (Atasheva *et al.*, 2014; Welsby *et al.*, 2014; Zhang, Y. *et al.*, 2015; Iwata *et al.*, 2016). Intriguingly, many of these enzymes, as well as PARP4 and PARP15, are under diversifying selective pressure in primates, suggesting an ADP-ribose “arms-race” between hosts and viral pathogens (Daugherty *et al.*, 2014).

With the recent development of better tools to detect ADP-ribose modification of proteins (Chang, 2018), many additional roles of ADP-ribosylation in a variety of cellular signalling pathways are likely to emerge in coming years.

### PARP1 and cell death

**Figure 3:**
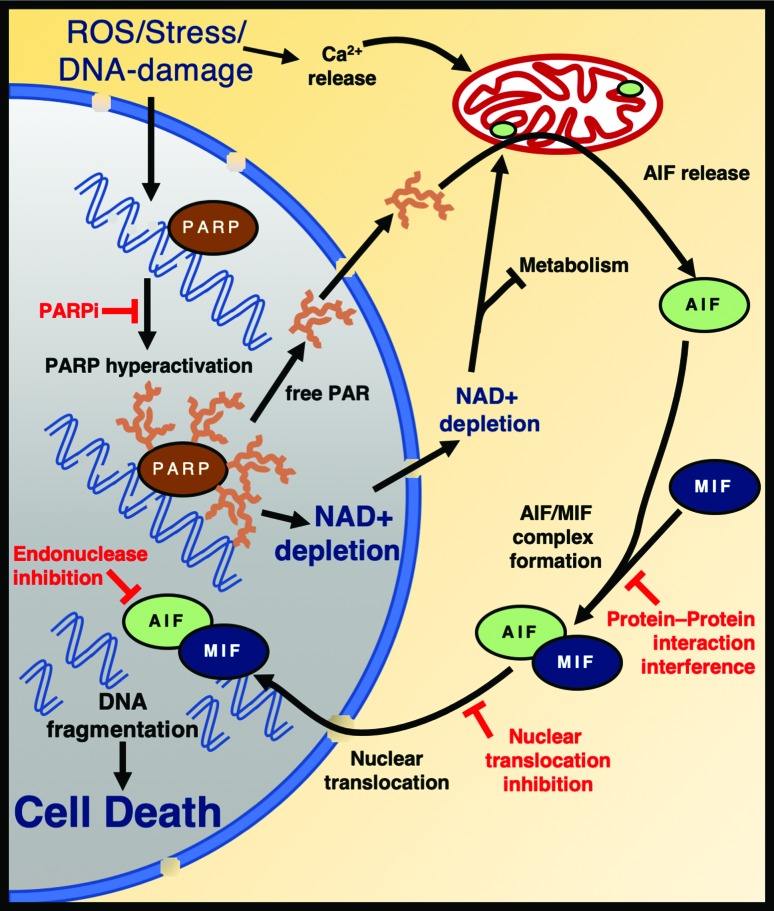
PARP1 mediates cell death by Parthanatos. Oxidative damage triggers PARP-hyperactivation, resulting in AIF release from the mitochondria and nuclear translocation of the AIF/MIF complex. Endonuclease activation causes cell death. Some of the potential therapeutic targets are depicted in red. The nucleus is coloured in grey and cytoplasm in orange. AIF: Apoptosis Inducing Factor. MIF: macrophage migration inhibitory factor.

Active PARP1 produces large amounts of PAR and at high levels of DNA damage up to 80% of the cellular NAD^+^ pool can be depleted within 5–15 min (D’Amours *et al.*, 1999). Since NAD^+^ is necessary for glyceraldehyde 3-phosphate dehydrogenase activity during glycolysis (Tan *et al.*, 2013), a reduction in NAD^+^ leads to lower pyruvate production, reducing carbon flow into the mitochondrial TCA cycle, and hence ATP production. Conversely, ATP is required for NAD^+^ synthesis, and therefore the uncontrolled use of NAD^+^ by PARP1 can lead to a bioenergetic collapse (Figure 3).

PARP1 hyperactivation initiates a programmed cell death pathway termed parthanatos, which is independent of canonical apoptosis, necrosis or autophagy (Yu *et al.*, 2002; Galluzzi *et al.*, 2018) and is mediated by the apoptosis-inducing factor (AIF) (Yu *et al.*, 2002; Andrabi *et al.*, 2006) (Figure 3). AIF is a mitochondrial membrane-anchored protein that is required for the assembly of the mitochondrial electron transport chain and exists in an equilibrium between monomeric and dimeric forms, with NAD(H) binding favouring dimer formation (Brosey *et al.*, 2016). Upon PARP1 hyperactivation, AIF is released from the mitochondria and translocates to the nucleus to drive parthanatos (Yu *et al.*, 2002; Otera *et al.*, 2005). How this occurs is currently unclear, but surprisingly, the AIF transmembrane fragment does not need to be cleaved (Wang, Y. *et al.*, 2009). One possibility is that NAD^+^ depletion itself promotes AIF release either by mitochondrial dysfunction (Alano *et al.*, 2010; Baxter *et al.*, 2014) or by inducing conformational changes in AIF (Sevrioukova, 2009; Brosey *et al.*, 2016). Alternatively, a direct interaction between AIF and protein-free PAR polymers has been suggested to mediate AIF release (Andrabi *et al.*, 2006; Wang, Y. *et al.*, 2011). Free AIF then promotes translocation of the nuclease MIF to the nucleus, which cleaves genomic DNA inducing cell death (Wang, Y. *et al.*, 2016a) (Figure 3). Many of the molecular mechanisms of parthanatos remain to be clarified, but an improved understanding of this pathway is critical for the development of novel treatment avenues for a potentially large number of diseases (see below).

## Human diseases and therapeutic opportunities

### PARP1/2 inhibition and HR-defective cancer

DNA-activated PARPs, particularly PARP1, became attractive drug target candidates for cancer therapy in 2005 when PARP inhibition (PARPi) was shown to induce synthetic lethality in cells lacking BRCA1/2 (Bryant *et al.*, 2005; Farmer *et al.*, 2005). As discussed above, in the absence of PARP1-dependent SSBR, unrepaired single-strand breaks are converted into DSBs by the passage of a replication fork, leading to a distinct requirement for BRCA1/2-dependent homologous recombination (HR) (Figure 2B). BRCA genes are tumour suppressors that are frequently mutated in breast and ovarian cancers, and four compounds (rucaparib, niraparib, olaparib and talazoparib) are currently licenced by the U.S. Food and Drug Administration (FDA) for treatment of BRCA-defective cancers (O’Connor, 2015; Bitler *et al.*, 2017). These inhibitors bind to the nicotinamide binding site in the catalytic domain, mimicking the three H-bonds established by the nicotinamide group from NAD^+^. By blocking PARP catalytic activity, these compounds slow single-strand break repair in two ways: a) the lack of PARylation surrounding break sites delays the recruitment of DNA repair factors such as XRCC1; and b) by preventing PARP1 auto-modification that is required for release of the protein from the DNA break (D’Amours *et al.*, 1999). Thus, these inhibitors lock or “trap” the enzyme bound to the DNA, preventing the access of other enzymes to the break (Bryant *et al.*, 2005; Pommier *et al.*, 2016; Lord and Ashworth, 2017). Novel inhibitors that induce more stable trapping of PARP seem to be better inducers of synthetic lethality in BRCA-mutated cells, suggesting that this trapping effect is crucial for PARP inhibitor efficacy (Murai *et al.*, 2014).

The clinical success of PARP inhibitors in BRCA1/2-mutated breast and ovarian cancers has ignited a push for more widespread use of these compounds in cancers with a molecular signature of defective HR, irrespective of which HR gene is mutated and in which tissue the tumour originated (Pilie *et al.*, 2019). Similarly, novel inhibitors that selectively target different PARPs, including PARP3, PARP5a/5b, PARP7, PARP10, PARP11 and PARP14 are under investigation for the targeted treatment of cancers with alterations in particular pathways (Ishida *et al.*, 2006; Lindgren *et al.*, 2013; Iwata *et al.*, 2016; Wang, Y. Q. *et al.*, 2016b; Ferri *et al.*, 2017; Yoneyama-Hirozane *et al.*, 2017; Kirby *et al.*, 2018; Moustakim *et al.*, 2018; Murthy *et al.*, 2018).

Remarkably, PARP1 inhibitors may also be of significant therapeutic value for non-oncological use both in rare neurological disorders in which excessive PARP signalling seems to be detrimental, as well as in more prevalent degenerative diseases in which parthanatos seems to play a central pathological role (Berger *et al.*, 2018) (discussed below).

### ADP-ribosylation in genetic neurodegenerative disorders

Mutations in single-strand break repair genes, such as PNKP, APTX, TDP1 and XRCC1, cause genetic neurodegenerative disorders characterised by severe cerebellar atrophy and ataxia (Moreira *et al.*, 2001; El-Khamisy *et al.*, 2005; Bras *et al.*, 2015; Hoch *et al.*, 2017). Treatment of cells from these patients with DNA damaging agents leads to excessive PARP1 activation, suggesting that defective single-strand break repair leads to overt signalling of these lesions (Hoch *et al.*, 2017). As deletion of PARP1 partially rescued many of the cerebellar defects observed in XRCC1-deficient mice, it was suggested that PARP1-induced parthanatos and/or NAD^+^ depletion contributes to disease pathology (Hoch *et al.*, 2017). Although PARP1 inhibition should in principle be beneficial in this scenario, the currently available PARP1 inhibitors are unlikely to be of therapeutic value, as the PARP1 trapping effect (discussed above) further compounds the DNA repair defect in these cells (Hoch *et al.*, 2017). In this context, inhibitors that better mimic genetic deletion of PARP1 would be desirable.

Mutations in enzymes involved in removing ADP-ribose modifications also leads to neurodegenerative disease, as illustrated by the identification of patients with mutations in the hydrolases ARH3 and TARG. ARH3 has specificity for both poly-ADP-ribose chains as well as mono-ADP-ribose moieties attached to serines (Abplanalp *et al.*, 2017; Fontana *et al.*, 2017), whereas TARG hydrolyses the ester linkage between mono-ADP-ribose and aspartate or glutamate side chains (Sharifi *et al.*, 2013). ARH3 mutations are associated with neurodegenerative defects such as ataxia and febrile seizures, while TARG1 loss causes severe developmental delay, epilepsy and quadriplegia (Sharifi *et al.*, 2013; Danhauser *et al.*, 2018; Ghosh *et al.*, 2018). Whereas TARG deficient cells shown signs of DNA-repair defects, a role for ARH3 in DNA damage responses is speculative at this point, although serine has been recently established as the primary acceptor of DNA damage-induced ADP-ribosylation (Palazzo *et al.*, 2018). If excessive PAR formation, NAD^+^ depletion and/or parthanatos are also involved in promoting the neurological defects seen in these patients, currently available catalytic PARP1 inhibitors may well be a viable therapeutic option (Danhauser *et al.*, 2018; Ghosh *et al.*, 2018).

Mutations in PARP10 lead to a neurodegenerative disorder associated with developmental delay and cortical atrophy, as well as delayed myelination (Shahrour *et al.*, 2016). Although a defect in PARP10-dependent Wnt or NFκB signalling was not determined, patient cells had a DNA repair defect in response to hydroxyurea (HU) and ultraviolet light (UV), and the pathology is reminiscent of other DNA repair disorders (Shahrour *et al.*, 2016). A more detailed understanding of the cellular consequences of PARP10 loss and which of its many functions is most important to prevent disease onset and progression will be critical to suggest possible therapeutic avenues for this disease.

### Parthanatos inhibition

PARP1-dependent cell death via parthanatos has been implicated in several critical pathological processes, such as ischemia-reperfusion injury in myocardial infarction and stroke, septic shock, brain trauma and neurodegenerative diseases such as Parkinsons disease and Alzheimers disease (Pacher and Szabo, 2007; Moroni, 2008; Lee *et al.*, 2013; Dawson and Dawson, 2017; Berger *et al.*, 2018; Henning *et al.*, 2018; Kam *et al.*, 2018; Zhang, J. *et al.*, 2018). A common theme among these disorders seems to be PARP1 hyperactivation in response to oxidative DNA damage, either as part of the reperfusion of oxygen-deprived tissues or caused by pathophysiological changes that induce the production of reactive oxygen species or nitric oxide.

Interestingly, PARP1 cytotoxicity seems to have a gender bias (McCullough *et al.*, 2005; Yuan *et al.*, 2009), with androgens promoting parthanatos (Vagnerova *et al.*, 2010; Sharma *et al.*, 2011), while oestrogens counteract it (Batnasan *et al.*, 2015). This raises the fascinating possibility that differential sensitivity to PARP1 hyperactivation might contribute to the higher male incidence of ischaemic stroke, sepsis and Parkinsons disease (Miller and Cronin-Golomb, 2010; Sakr *et al.*, 2013; Barker-Collo *et al.*, 2015).

Mounting pre-clinical evidence suggests that PARP1 knockout or PARP inhibitor treatment have profound beneficial effects in mouse models of parthanatos-induced pathologies, preventing cell death and tissue dysfunction (Pacher and Szabo, 2007; Dawson and Dawson, 2017; Berger *et al.*, 2018; Henning *et al.*, 2018). These results have prompted calls for clinical trials to repurpose PARP inhibitors for the treatment of these disorders, particularly when no other viable treatment option exists (Berger *et al.*, 2018). Conceptually, targeting other steps in parthanatos, such as preventing mitochondrial AIF release or inhibiting nuclear import or activation of the MIF nuclease may also be of therapeutic value (Figure 3). Although these compounds would have to undergo extensive pre-clinical and clinical efficacy and safety trials, their development may be warranted by a reduced potential for DNA repair-associated side-effects of systemic PARP inhibition during chronic treatment.

## Concluding remarks

Detailed knowledge of the processes and pathways regulated by post-translational modifications such as phosphorylation and ubiquitination led to the development of a myriad of kinase inhibitors and molecules targeting the ubiquitin system, either already in clinical use or in clinical trials (Ferguson and Gray, 2018; Wertz and Wang, 2019). In recent years, novel tools to study ADP-ribosylation have allowed a rapid development in this field, characterising many of the “writers”, “erasers” and “readers” of this modification. Taken together with the fact that the first PARP inhibitor only entered the clinic in 2014, this raises the exciting prospect that a more detailed understanding of ADP-ribose metabolism, particularly of less well-studied PARPs and hydrolases, may well yield novel therapeutic strategies in coming years.
